# Unveiling Fungal Community Structure along Different Levels of Anthropic Disturbance in a South American Subtropical Lagoon

**DOI:** 10.3390/jof9090890

**Published:** 2023-08-31

**Authors:** Danielle Machado Pagani, Stefânia P. R. Ventura, Duong Vu, Thairine Mendes-Pereira, Luiz Marcelo Ribeiro Tomé, Daniel Santana de Carvalho, Diogo Henrique Costa-Rezende, Rodrigo Bentes Kato, Glen Jasper Yupanqui García, József Geml, Vincent Robert, Ng Haig They, Bertram Brenig, Vasco Azevedo, Maria Lúcia Scroferneker, Patricia Valente, Aristóteles Góes-Neto

**Affiliations:** 1 Programa de Pós-Graduação em Microbiologia Agrícola e do Ambiente, Universidade Federal do Rio Grande do Sul, Porto Alegre 90010-150, RS, Brazil; daniellepagani@gmail.com (D.M.P.); patricia.valente@ufrgs.br (P.V.); 2 Programa de Pós-Graduação em Bioinformática, Universidade Federal de Minas Gerais, Belo Horizonte 31270-901, MG, Brazil; stefania.ventura2@gmail.com (S.P.R.V.); rbkato@gmail.com (R.B.K.); glen.yupanqui@gmail.com (G.J.Y.G.); vascoariston@gmail.com (V.A.); 3 Westerdijk Fungal Biodiversity Institute, Uppsalalaan 8, 3584 CT Utrecht, The Netherlands; d.vu@wi.knaw.nl (D.V.); v.robert@wi.knaw.nl (V.R.); 4 Programa de Pós-Graduação em Microbiologia, Universidade Federal de Minas Gerais, Belo Horizonte 31270-901, MG, Brazil; thairinemp@gmail.com (T.M.-P.); marcelofsa_rt@hotmail.com (L.M.R.T.); danielscarv@gmail.com (D.S.d.C.); 5 Departamento de Ciências Biológicas, Programa de Pós-Graduação em Botânica, Universidade Estadual de Feira de Santana, Feira de Santana 44036-900, BA, Brazil; diogobio.dh@gmail.com; 6 ELKH-EKKE Lendület Environmental Microbiome Research Group, Eszterházy Károly Catholic University, Leányka U. 6, 3300 Eger, Hungary; jozsef.geml@gmail.com; 7 Laboratório de Ecologia Aquática Microbiana, Programa de Pós-Graduação em Biologia de Ambientes Aquáticos Continentais, Departamento Interdisciplinar, Centro de Estudos Costeiros, Limnológicos e Marinhos, Universidade Federal do Rio Grande do Sul, Campus Litoral Norte, Tramandaí 95590-000, RS, Brazil; haigthey@ufrgs.br; 8 Institute of Veterinary Medicine, Georg-August-University Goettingen, 37073 Göttingen, Germany; bbrenig@gwdg.de; 9 Laboratory of Cellular and Molecular Genetics, Instituto de Ciências Biológicas, Universidade Federal de Minas Gerais, Belo Horizonte 31270-901, MG, Brazil; 10 Programa de Pós-Graduação em Ciências Médicas, Universidade Federal do Rio Grande do Sul, Porto Alegre 90010-150, RS, Brazil; scrofern@ufrgs.br

**Keywords:** freshwater, fungi, amplicon metagenomics

## Abstract

Studies of fungal communities through amplicon metagenomics in aquatic environments, particularly in freshwater ecosystems, are still relatively recent. Unfortunately, many of these water bodies are facing growing threats from human expansion, such as effluent discharge from various human activities. As a result, these effluents have the potential to significantly alter the characteristics of water bodies and, subsequently, impact the diversity of their resident microorganisms. In this context, our objective was to investigate whether the fungal community structure varies according to the presence of different anthropic disturbances. We expect (i) the diversity of fungi will be greater and (ii) more specific unique operational taxonomic units (OTUs) related to each ecotonal system will be found compared to other sites of a lagoon. The study was conducted in the Tramandaí Lagoon (subtropical southern Brazil) at four distinct sampling points (estuary, middle of the lagoon, crop field area, and near a residential area where the Tramandaí River flows into the lagoon). As expected, the estuary and residential zones, which are ecotones, exhibited greater fungal diversity and more specific OTUs compared to the middle of the lagoon and crop field area. Moreover, a substantial proportion of fungal taxa could not be identified at the genus level, with many only classified at the phylum level, indicating potential new lineages. These findings underscore our limited understanding of the subtropical freshwater mycobiota.

## 1. Introduction

Freshwater ecosystems are vital as they provide habitats for nearly 9.5% of all described species [1,2]. These ecosystems are distributed worldwide and exist in various forms, including rivers, lakes, swamps, ponds, and streams. Nevertheless, the rapid deterioration of these water bodies due to human activities, driven by population growth, poses a significant threat to the biodiversity of aquatic habitats [3]. This detrimental impact extends to microorganisms, such as fungi, which are key players in these environments even though they are highly understudied [1].

It was once believed that fungi were not as diverse or abundant in aquatic ecosystems as compared to terrestrial ones [4]. Nonetheless, recent studies conducted in various freshwater and marine habitats have shown that fungi can be a significant part of the eukaryotic community in aquatic environments [5,6,7], even reaching a biomass comparable to that of prokaryotes in highly productive offshore systems [8]. In aquatic ecosystems, fungi play fundamental functional roles, such as leaf decomposers, in rivers, mangroves, and wetlands [9], as well as acting as parasites and saprophytes in lacustrine ecosystems [6,10]. Nonetheless, their high local diversity and substrate heterogeneity make it difficult to assess spatial and temporal changes in their diversity, especially on large spatiotemporal scales. Furthermore, the limitations of traditional culture-dependent or direct examination approaches may also highly underestimate fungal diversity and complexity.

Recently, the analysis of fungal DNA in freshwater rivers and lakes has shown the possibility of efficiently assessing fungal diversity in ecosystems and highlighted gaps in our understanding of their phylogeny [5]. This method enables the detection of taxa presence without relying on culture-dependent or direct examination approaches. It utilizes indicators such as enzymes, feces, and epidermal cells, which are left behind by organisms, specifically for fungi, spores, and fragments of mycelia [11,12]. Additionally, the use of molecular-based detection of genomic markers can also provide insights into the functional roles of fungi in aquatic ecosystems, such as their roles in nutrient cycling and carbon sequestration [13]. Although molecular techniques have been frequently used to estimate the diversity of fungi (and other microorganisms) in different locations [14,15,16], these studies are still rare in tropical and subtropical freshwater environments [5]. The subtropical region is a transitional zone between tropical and temperate climates, characterized by cold and usually dry winters and rainy and moderate temperatures in summer [17]. Subtropical areas can be found in various continents, such as the Mediterranean region, the southern United States, parts of Australia, South Africa, Asia, and South America (as in southern Brazil) [18].

Tropical and subtropical freshwater environments are known for their high levels of biodiversity and unique ecological niches. Despite advances in sequencing technologies, there still are, however, few studies estimating fungal diversity in those regions [19]. As a result, 96% of all fungal taxa have been recorded in temperate regions, with fewer recorded in tropical and subtropical regions [20]. Moreover, human habitat modification and pollution are drastically impacting microbial diversity in tropical and subtropical regions to an unknown extent since few previous data are available. In lagoons, it is common to receive daily discharges from rivers and the sea, making them transition zone ecosystems, often known as ecotones [21]. Apart from these contributions, they can also receive localized and diffuse sources of effluents of domestic and industrial origins. Consequently, these distinct local contributions can create different environments in the same lagoon (spatial heterogeneity), resulting in alterations in the components of aquatic ecosystems. As fungal diversity and abundance are affected by the physical and chemical conditions of the water [22], it is expected that the diversity of fungal communities will vary depending on the degree of human impact.

In this study, we conducted a metabarcoding survey to evaluate the fungal community structure across a South American lagoon with spatial heterogeneity in local discharges. Our objective was to investigate whether the fungal community structure varies according to the presence of different anthropic disturbances, in terms of species diversity, community structure, and composition. Specifically, we anticipated (i) the diversity of fungi would be greater and (ii) more specific unique taxa related to each ecotonal system (close to the lagoon and river mouths) would be found compared to other sites of the lagoon. Consequently, we expected that beta diversity would differ among areas.

## 2. Materials and Methods

### 2.1. Study Area

We collected water samples from the Tramandaí Lagoon, Rio Grande do Sul state, Brazil (29°58′12.59″ S; 50°9′19.72″ W) [23]. This lagoon is part of the Tramandaí hydrographic basin and has a surface area of 18.8 km². Uses of the Tramandaí Lagoon water include irrigation of rice, activities related to summer holidaymaking, public supply, and dilution of domestic and industrial wastes, particularly near urban areas [24]. These activities have the potential to cause significant impacts by negatively altering the hydraulic attributes of the system and affecting its biodiversity.

**Figure 1:**
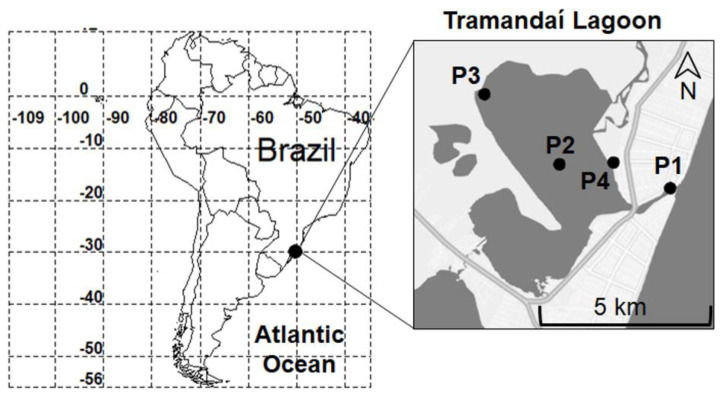
Location of the study area and sampling points of the Tramandaí Lagoon. P1 was situated inside an estuary, P2 was in the middle of the lagoon without marginal anthropogenic pressure, P3 was in a non-urbanized area and surrounded by native forest with nearby crop fields, and P4 was near a residential area where the Tramandaí River flows into the lagoon.

Sampling within the Tramandaí Lagoon was conducted during the ebbing tide [25]. Sampling points were selected according to types of anthropic disturbance (Figure 1). Sampling point 1 (29°58′34.7″ S 50° 07′ 18.8″ W) was situated at the estuarine mouth; sampling point 2 (29° 58′12.2” S 50°09′19.7” W) was in the middle of the lagoon without any direct anthropogenic pressure; sampling point 3 (29°57′05.8″ S 50°10′41.7″ W) was in a non-urbanized area, surrounded by native forest with nearby crop fields; and sampling point 4 (29°58′10.4″ S 50°08′20.7″ W) was most protected from marine currents, located closest to a connection with the Tramandaí River through which water flows from other locations in the Basin to enter the Tramandaí Lagoon, and was in a densely urbanized zone (Figure 1).

### 2.2. Sampling Design and Collection

In each sampling area (P1–P4), a total of three sampling units, spaced at a distance of approximately 1 m, were obtained and named S1, S2, S3 for P1; S4, S5, S6 for P2; S7, S8, S9 for P3; and S10, S11, S12 for P4; totaling 12 sampling units. The distance among the points was at least 3 km from P1 to P4 and from P2 to P3; 1.56 km from P2 to P4; and 7 km from P3 to P1. In addition to sample collection for analyzing fungal communities, water samples were collected at the water subsurface (5–10 cm). A total of 1050 mL per sampling area (350 mL per sampling unit) was collected during the summer (March 2019).

Water temperature (°C), pH, and conductivity (μs/cm) were measured using a portable multiparametric (PHOX P50 and C50) probe during the collections. Initial dissolved oxygen (mg L^−1^) and biological oxygen demand (mg L^−1^) were measured in the laboratory using the Winkler (iodometric) method [26]. Chloride concentration (mg L^−1^) and salinity (‰) were also measured in the laboratory using the argentometric method [26].

### 2.3. eDNA Extraction, Amplification, and High-Throughput Sequencing

A total of 100 mL subsample was filtered using a 0.22 µm membrane (Millipore), in triplicate, and the filtrate was submitted to eDNA extraction using the DNeasy PowerSoil Kit (QIAGEN). The quality and quantity of eDNA were evaluated using spectrophotometry (NanoDrop ND 1000, NanoDrop Technologies, Wilmington, DE, USA). For the metabarcoding approach, PCR was performed using the primers fITS7 (5’-GTGARTCATCGAATCTTTG-3’) [27] and ITS4 (5’-TCCTCCGCTTATTGATATGC-3’) [28], which target the ITS2 region of the fungal nuclear ribosomal DNA. While the selection of a specific genomic region might lead to the targeting of a particular group of fungi, the use of ITS markers stands out for yielding a significantly higher number of reads. Therefore, the increased read counts compensate for the use of this region when studying fungal communities [29]. PCR amplification was performed as follows: 94 °C for 2 min, followed by 35 cycles of denaturation at 94 °C for 1 min, annealing at 60 °C for 1 min, extension at 72 °C for 3 min, and a final extension step at 72 °C for 5 min. At least three independent amplification reactions were performed from the same DNA extraction to account for the stochasticity. PCR products were then pooled in equimolar proportions based on their DNA concentrations and purified using AMPure beads.

The DNA was quantified using a fluorescence assay using Qubit 2.0 Fluorometer (Thermo, Waltham, MA, USA) and Qubit dsDNA BR Assay Kit (Thermo, Waltham, MA, USA). Sequencing libraries were generated using a TrueSeq DNA PCR-Free Sample Preparation Kit (Illumina, San Diego, CA, USA), following the manufacturer’s recommendations, and index codes were added. The library quality was assessed using a Qubit@ 2.0 Fluorometer (Thermo Scientific, Waltham, MA, USA) and the Bioanalyzer 2100 system (Agilent, Santa Clara, CA, USA), and then sequenced on a HiSeq 2500 platform (Illumina, San Diego, CA, USA). All raw generated sequences were deposited in NCBI SRA under BioProject ID: PRJNA991127.

### 2.4. Bioinformatic Analyses

The output files (FASTQ format) of the metabarcoding sequencing of each sample comprised our raw primary data. The bioinformatics pipeline was elaborated and run on an Operational System Ubuntu 16.04.5 LTS system. The following programs were used: VSEARCH v2.9.1 [30] and BLAST v2.2.31 [31]. Scripts in Bash [32] and Python v3.0 [33] programming languages were written to automate some tasks, such as merging samples or generating the abundance table. The reference database used for fungal taxonomic identification was UNITE v. 9.0 [34]. The pipeline comprised the following steps: (i) quality and length filtering was performed with VSEARCH, with sequences smaller than 300 bp removed and default settings for quality filtering; (ii) dereplication was performed with VSEARCH; (iii) detection and removal of chimeric sequences was performed using the UNITE database (uchime_reference_dataset_untrimmed.fasta) and de novo implementation was performed with VSEARCH; (iv) clustering sequences with similarity above 97% was performed with VSEARCH; (v) taxonomic classification of OTUs was performed with the SINTAX algorithm [35], which is part of USEARCH [36], and the fungal taxonomic database UNITE UCHIME reference dataset v 9.0 [34], and consolidation of the table of OTU abundance with taxonomic classifications was performed using the get_abundances_table_asv.py script included in the source code of the pipeline for amplicon analysis deposited on our lab GitHub: https://github.com/LBMCF/pipeline-for-amplicon-analysis, accessed on 30 January 2023. Trophic mode, ecological guilds, and body morphology were annotated to OTUs identified at the genus level with FUNGuild [37], using the FUNGuild.py script (https://github.com/UMNFuN/FUNGuild, accessed on March 2023) (Table S1). After analyzing the taxonomic abundance of the OTUs, we selected the 10% most dominant OTUs (n = 193) to perform phylogenetic analysis, complex network analysis, and canonical correspondence analysis (CCA) in order to improve the interpretation of the results.

### 2.5. Phylogenetic Analyses

As less than 37.5% of sequences were identified to any infrakingdom taxonomic level using BLAST, we used the phylogenetic tree topology to verify the closer taxa and improve the taxonomic attribution of putative phylum for each OTU. Representative sequences of the recovered OTUs (n = 1930) were used for the phylogenetic analysis. The alignment of the ITS2 region was performed using Geneious Prime v. 2022.1.1 [38] with the MUSCLE algorithm. The final alignment length was 5891 bp, comprising 3984 parsimony-informative and 898 singleton sites. For selecting the evolutionary model for the phylogenetic analysis, we used ModelFinder [39], which selected the TIM + F + G4 model according to the Bayesian Information Criteria (BIC score: 1232018.287). Maximum likelihood analysis was performed with IQ-TREE multicore v. 1.6.12 [40], generating 1000 bootstrap iterations. The consensus tree was visualized using FigTree v. 3.5.9 [41]. Numerical values on branches indicate bootstrap percentages (>50%) and colors on tips indicate the phylum of the sequences identified using BLAST. Branches including OTUs that were not identified at any taxonomic level were collapsed at the tree to improve the visualization of the nodes.

The maximum likelihood phylogenetic analysis of the 10% most dominant OTUs (n = 193) was generated from a final alignment length of 1472 bp. We used the GTR + F + I + G4 evolutionary model to perform 1000 bootstrap iterations, using the same criteria as the former analysis.

### 2.6. Community Ecology Analyses

Ecological and statistical analyses were performed using R v. 4.3.0 [42] or Python customized scripts and Microbiome Analyst [43,44] to assess the taxonomic composition, richness, and abundance of probable species (OTUs). Sampling sufficiency was evaluated by generating rarefaction curves by samples and by OTUs, and read count data were centered and log-ratio transformed before all statistical analyses [45]. Subsequently, the following analyses were performed to jointly analyze the microbiome: alpha and beta diversities [43], co-occurrence analyses using complex networks [46], and canonical correspondence analyses (CCA).

#### 2.6.1. Alpha Diversity

Alpha diversity values, based on the Shannon index at OTU taxonomic level, were calculated for the distinct areas (P1, P2, P3, and P4). Differences among areas were tested with ANOVA. All alpha diversity analyses were performed based on scripts and packages in R software v. 4.3.0 [42], and data visualization was produced using the package ggplot2 in R.

#### 2.6.2. Beta Diversity

Pairwise beta diversity among areas was calculated with the Bray-Curtis index of distance at the OTU taxonomic level. Differences in OTU composition among areas were tested with permutational multivariate analysis of variance (PERMANOVA) [47]. In order to test if groups of samples had a difference in intragroup community variation, also known as heterogeneity of multivariate dispersion, the PERMDISP2 procedure was used [48]. Additionally, we also ran non-metric multidimensional scaling (NMDS) using the Bray–Curtis index. All beta diversity analyses were performed based on scripts and packages in R software v. 4.3.0 [42], and data visualization was performed using the package ggplot2 in R.

#### 2.6.3. Complex Network Analyses

We constructed two co-occurrence networks, one with all OTUs (n = 1930) and another with the 10% most dominant OTUs (n = 193). The co-occurrence networks were generated considering each OTU as a node. The nodes were connected whenever two or more OTUs co-occurred in the same environment. The node size is proportional to the number of environments in which the OTU occurred. For instance, larger nodes represent OTUs that are present in more environments. The colors represent the environments and different combinations of environments in which an OTU occurred. The software Gephi v. 0.9.2 was used to plot networks [46].

#### 2.6.4. Canonical Correspondence Analyses

The two canonical correspondence analyses (CCAs), one with all OTUs and another with the 10% most dominant OTUs (n = 193) were implemented in PAST v. 4.08 [49] in accordance with the eigenanalysis algorithm [50]. The ordinations were given as site scores, and physicochemical variables (salinity, pH, conductivity, total dissolved chlorides, dissolved oxygen, biological oxygen demand, and water temperature) were plotted as correlations with site scores.

## 3. Results

### 3.1. Taxonomic Composition

**Figure 2:**
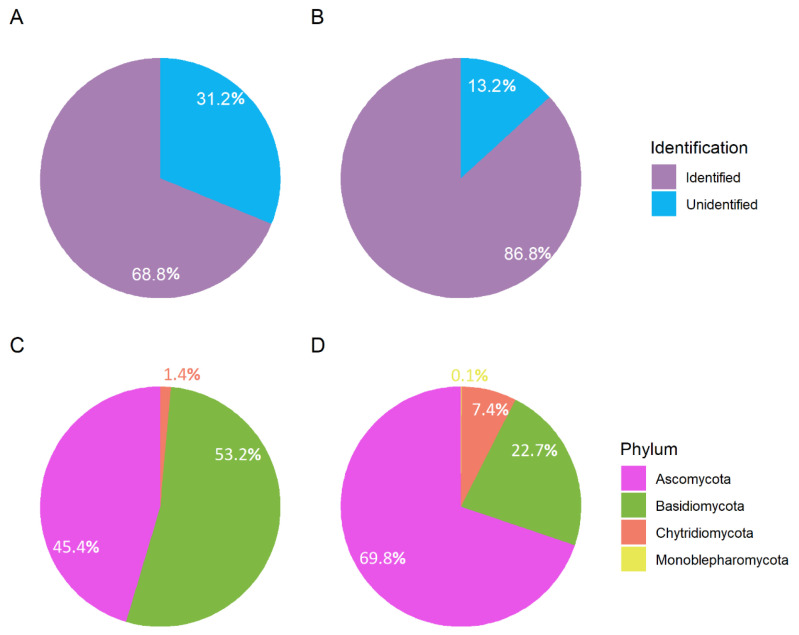
Relative proportions (**A**) between unidentified and identified reads and (**B**) between unidentified and identified putative fungal species (OTUs) in the Tramandaí Lagoon, Brazil. Phylum relative proportions (**C**) between reads identified and (**D**) between OTUs identified.

A total of 788,365 reads were sequenced, but only 68.8% could be confidently assigned to any infrakingdom taxonomic levels (Figure 2A). When considering only the relative abundance assigned for each phylum in the assigned infrakingdom levels, Basidiomycota represented 53.2%, while Ascomycota represented 45.4%, and the other fungal phyla accounted for only 1.4% of the reads (Figure 2C).

**Figure 3:**
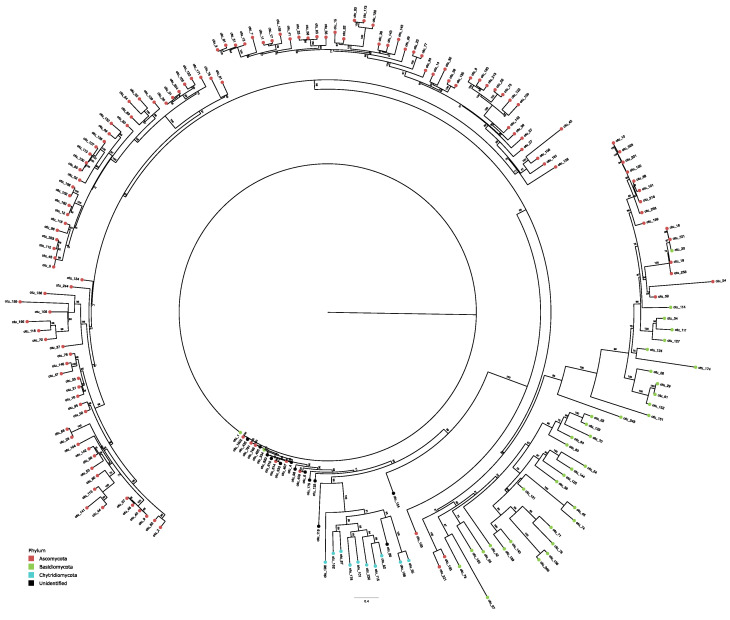
Maximum likelihood phylogenetic tree using the 10% most dominant OTUs retrieved (n = 193 OTUs) in all sampling points, generated with 1000 bootstrap replicates. Node tips are identified at the three phyla recovered: Ascomycota (orange), Basidiomycota (green), and Chytridiomycota (blue). Monoblepharomycota is not included in this phylogeny since it is underrepresented.

Additionally, a total of 1930 putative fungal species (OTUs) were identified in all sampling points. Nonetheless, approximately only 37.5% of sequences were identified to any infrakingdom taxonomic level. After using the phylogenetic tree topology to verify the closer taxa (Figure S1), we greatly improved the taxonomic classification to 86.8% assigned to any infrakingdom taxonomic levels (phylum, class, order, family, or genus) (Figure 2B). The majority of identified OTUs comprised Ascomycota (69.8%), whereas Basidiomycota encompassed 22.7%, Chytridiomycota encompassed 7.4%, and Monoblepharomycota accounted for 0.1% (Figure 2D). Phylogenetic analysis of the 10% most dominant OTUs showed that only Ascomycota, Basidiomycota, and Chytridiomycota were among the most dominant taxa (Figure 3).

Additionally, a total of 84 fungal genera were identified (43 in Ascomycota, 39 in Basidiomycota, and 2 in Chytridiomycota). Most were saprotrophs and/or patotrophs with mycelial body morphology, occurring in both terrestrial and aquatic environments [37] (Table 1).

The three most prevalent (occurring in all areas) and abundant taxa (with a total number of reads > 50,000) (OTUs: 1, 4, and 6) could not even be assigned at the subphylum level using BLAST. Furthermore, for those OTUs that could be identified at the genus level, the Ascomycota *Metarhizium* and *Trichoderma* were the most prevalent and abundant identified fungal genera. Specifically, 62.2% of *Metarhizium* were found in P1, 31.3% in P4, 5% in P3, and 1.5% in P2. Regarding *Trichoderma*, 51.8% were registered in P4, 27.3% in P3, 14% in P1, and 6.5% in P2. Meanwhile, *Schizophyllum* was the most abundant and prevalent Basidiomycota genus, considering that 68.4% were found in P1, 23.2% in P4, 6.6% in P3, and 1.8% in P2.

### 3.2. Alpha Diversity

**Figure 4:**
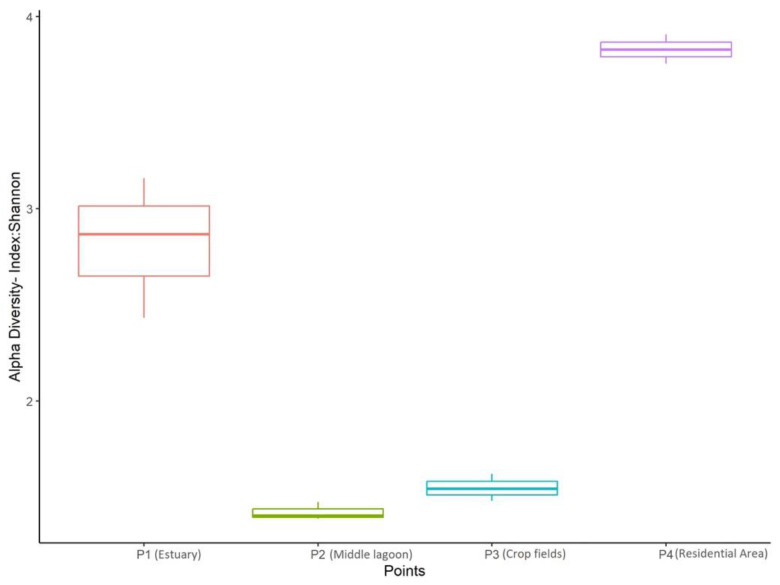
Boxplot representing each sampling point with all samples (three per point). Shannon diversity index values are depicted on the vertical axis while the distinct sampling points are shown on the horizontal axis (ANOVA significant difference at *p* < 0.05).

The Shannon diversity index values of the three sampling points were significantly distinct (ANOVA, *p* < 0.05). The residential area (P4) was the most diverse, followed by the estuary mouth (P1). The middle of the lagoon (P2) and crop field area (P3) exhibited significantly lower Shannon diversity (Figure 4).

### 3.3. Beta Diversity

**Figure 5:**
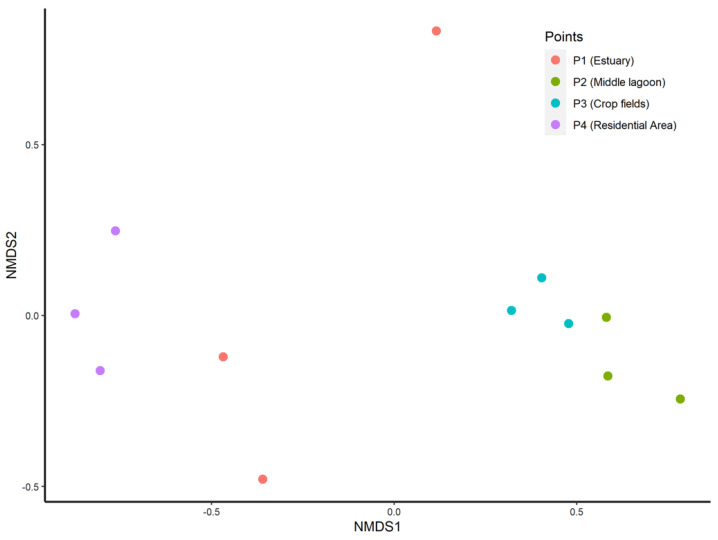
Characterization of beta diversity by non-metric multidimensional scaling (NMDS) plot of different environments of the Tramandaí Lagoon (stress = 0.05). For each environment, a total of three sampling units were obtained. Each circle represents one sampling unit, and each distinct color represents an environment. (PERMANOVA: PERMANOVA: F_(3,8)_ = 3.61, *p* < 0.5).

Beta diversity profiling by NMDS (stress = 0.05) displayed sampling units of the same area grouped together, and axis 1 was the main determinant for the ordination of the areas (Figure 5). Moreover, there was a significant difference (PERMANOVA: F_(3,8)_ = 3.61, *p* < 0.5) among the four areas (P1, P2, P3, and P4) of the Tramandaí Lagoon. Additionally, this difference in composition between the points did not occur due to a difference in intragroup community variation (PERMDISP: F_(3,8)_ = 1.79, *p* = 0.22).

### 3.4. Co-Occurrence Networks

**Figure 6:**
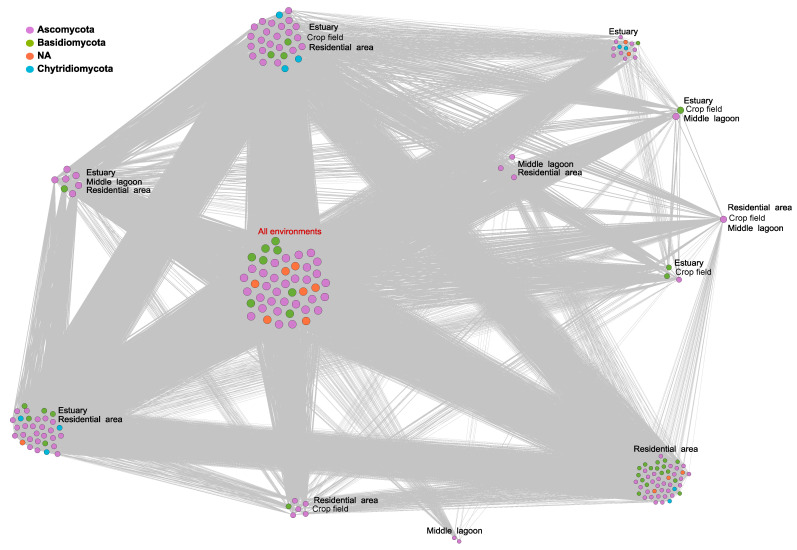
Co-occurrence network of putative species (OTUs) in the sampling areas (estuary, middle of the lagoon, residential area, and crop field area). Nodes represent the 10% most dominant OTUs, and edges indicate that OTUs co-occur in two or more sampling areas. Node size is proportional to the number of sampling areas in which such OTU occurred. The phyla Ascomycota, Basidiomycota, and Chytridiomycota are represented, and NA represents unidentified fungi.

The co-occurrence network of the 10% most dominant OTUs in the distinct sampling areas had order (N) 193 nodes and size (M) 17,264 edges. The average degree <k> = 177.264, the average clustering coefficient C = 0.958, and modularity md = 0.07 (Figure 6). The strictest core mycobiome community, i.e., those OTUs that co-occurred in all samples in all areas, comprised 46 distinct OTUs (Table S2), mainly Ascomycota, Basidiomycota, and unidentified fungi. The residential area (P4), followed by the estuary (P1), exhibited most of the unique OTUs, 50 and 14, respectively. On the other hand, the middle of the lagoon (P2) exhibited 2 unique OTUs, and the crop field area (P3) did not exhibit unique OTUs (Figure 6).

The co-occurrence network of all OTUs had order (N) 1930 nodes and size (M) 1,883,862 edges (<k> = 1.952; C = 0.944; md = 0.322; Figure S2). The strictest core mycobiome community comprised 61 distinct OTUs (Table S3), mainly Ascomycota, Basidiomycota, and unidentified fungi. The estuary (P1) exhibited most of the unique OTUs (n = 815), followed by the residential area (P4) (n = 602). The crop field area (P3) exhibited 123 unique OTUs, and the middle of the lagoon (P2) exhibited 108 unique OTUs (Table S3).

### 3.5. Canonical Correspondence Analyses

**Figure 7:**
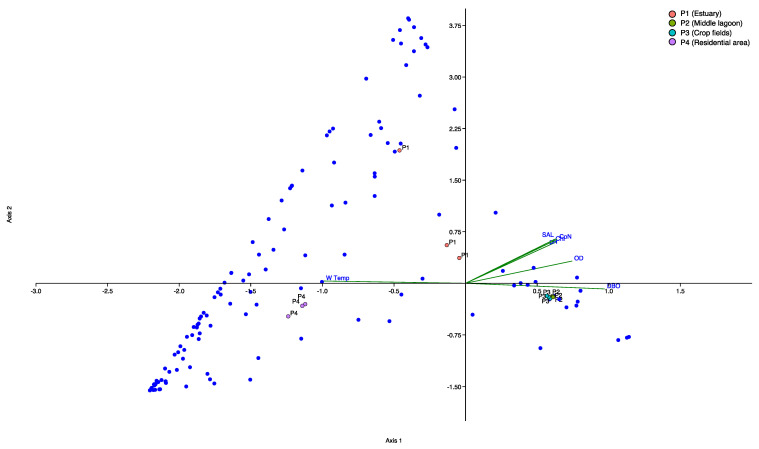
Integrative mycobiome and physicochemical analysis of the four sampling areas of the Tramandaí Lagoon by canonical correspondence analysis (CCA). Black dots are sampling areas, blue dots are the 10% most dominant OTUs, and green vectors are environmental variables. The P2 and P3 samples overlapped near axis 1, and the pH, salinity, total chlorides, and conductivity variables also showed overlap. The identification of OTUs (blue dots) is not depicted in order to maintain the clarity of the figure but can be consulted in Table S1.

Both CCAs demonstrated that the physicochemical variables influenced species composition. Regarding the CCA for only the 10% most abundant OTUs (n = 193), we observed that the CCA was significant (pseudo-F = 2.30, *p* = 0.001), and the constraining variables explained 0.5% of the total inertia (unadjusted R2). The first (53.2%) and second (24.6%) canonical axes accounted for 77.8% of the total constrained variance explained. Up to one axis was significant at α = 0.05, CCA1: pseudo-F = 4.68; *p* = 0.001. The variables with the highest contribution per axis in decreasing importance (higher loadings) were as follows: axis 1: BOD, DO, and pH (Figure 7).

In relation to the CCA for all OTUs, the CCA was significant (pseudo-F = 2.58, *p* = 0.01), and the constraining variables explained 0.5% of the total inertia (unadjusted R2). The first (68.4%) and second (30.4%) canonical axes accounted for 98.4% of the total constrained variance explained. Up to two axes were significant at α = 0.05, CCA1: pseudo-F = 5.31; *p* = 0.001, and CCA2: pseudo-F = 2.36, *p* = 0.05. The variables with the highest contribution per axis in decreasing importance (higher loadings) were as follows: axis 1: BOD, DO, and pH, and axis 2: pH, DO, and BOD (Figure S3).

## 4. Discussion

We used a metabarcoding approach to demonstrate that the fungal communities in the Tramandaí Lagoon exhibited variations based on the major direct environmental and anthropic influences. During the study period, we identified 1930 fungal OTUs in the lagoon. As expected, the estuary (P1) and residential (P4) zones, which are ecotones, exhibited greater fungal diversity than the middle of the lagoon and crop field area. Nonetheless, it is worth noting that despite 86.8% of the OTUs being identified at the phylum level, which was similar to other studies [51,52], only 16.2% of these were identified at the genus level, indicating that even with the advances in metabarcoding techniques, this method is relatively new and several taxa are still underrepresented in databases. As such, a large number of unknown fungi may be undetected. Thus, the fungal community of subtropical water bodies is still largely unknown. Without identifying these fungi, it is not possible to determine their role in the environment or the extent of their response to anthropogenic impacts.

The Tramandaí Lagoon displays different microenvironments in the same lagoon. Nevertheless, we observed the dominance of Ascomycota, Basidiomycota, and Chytridiomycota fungi, which was consistent with previous studies in freshwater environments [10,52,53]. Fungi found in aquatic environments can be dependent on this habitat throughout their entire life cycle or at least part of it. Based on their degree of adaptation, activity, and dependence on aquatic habitats, these fungi can be categorized into three groups [54]: (i) residents, comprising well-adapted fungi that are consistently active in aquatic environments; (ii) periodic immigrants, encompassing less adapted fungi that are only periodically active in aquatic environments, and (iii) versatile immigrants, which are poorly adapted fungi that are only sporadically active in aquatic habitats [5]. The genera *Metarhizium* and *Trichoderma* were the most abundant and prevalent Ascomycota in our study, whereas *Schizophyllum* was the most abundant and prevalent Basidiomycota genus. Both Ascomycota genera are soilborne fungi used as biocontrol agents [55,56,57]. These fungi produce a high load of spores and are commonly used as suspensions for several crops, which can be washed from the soil surface and carried into adjacent rivers and lagoons. Since these organisms were present at all points of the lagoon, but in a larger proportion at points P1 and P4, they could enter the lagoon through these ecotone zones. Notably, P3 did not exhibit the highest *Metarhizium* and *Trichoderma* abundance. This suggested that a small portion of the waste originating from crop fields could be deposited in the lagoon, while the majority could be deposited into other rivers in the region. As a result, these waste materials indirectly reach the lagoon through the region’s rivers, which could explain the heightened presence and prevalence of *Metarhizium* and *Trichoderma* in ecotone regions. The Tramandaí River runs through a large area that includes regions near rice and other crops, so it is possible that it transports a substantial volume of pesticides used in these agricultural activities into the lagoon [23,58]. The genus *Schizophyllum* comprises species with a wide distribution that are often parasites of trees, but they mainly adopt a saprobic lifestyle causing white rot [59,60]. The spores of this fungus are likely carried to the lagoon either by rain or the river that flows into it. This could explain the fact that we observed a higher proportion of these organisms at points P1 and P4, which are ecotone areas subject both to marine and fluvial influences. Therefore, the most abundant and prevalent genera of fungi in the Tramandaí Lagoon were versatile immigrants carried by rain, rivers, or sewage into the lagoon.

Nearly 8% of the identified OTUs belonged to the phylum Chytridiomycota, which are commonly considered resident fungi of aquatic environments due to their production of flagellated and motile zoospores [4,61,62]. These spores can move in water and moist soil, enabling them to spread to new food sources or hosts. Perhaps due to their reliance on water, we observed a higher abundance of fungi from this phylum at point P4 of the lagoon, which is under the influence of the sea and rivers in the region [24,25]. Consequently, these organisms might originate from various locations and find their way into the lagoon through rivers or the sea. This phylum plays a crucial ecological role, particularly in aquatic ecosystems, where they are involved in decomposing organic materials and cycling nutrients [4]. Among the Chytridiomycota found in the Tramandaí Lagoon, the identified OTUs belonged to the orders Chytridiales and Rhizophlyctidales. The former encompasses cosmopolitan organisms that exhibit a wide distribution across aquatic, continental, marine, and even moist terrestrial environments [4]. These organisms can be found engaged in saprophytic lifestyles or parasitizing various biological entities, such as algae, microscopic animals, other fungi, and amphibians [63]. Rhizophlyctidales, in turn, are typically found in freshwater habitats and are known for parasitizing algae and aquatic plants [64]. Members of this order are characterized by the presence of a rhizomycelial system, which helps them anchor to their host and absorb nutrients [64]. This supports our results of having encountered organisms of this order in a higher proportion at point P4, in an area near the Tramandaí River [24,25]. Rhizophlyctidales play an essential role in the ecology of freshwater ecosystems by regulating the growth of algae and aquatic plants [64]. They also serve as a food source for many aquatic animals, including insects, fish, and crustaceans. It is important to highlight that a significant proportion of Chytridiomycota OTUs were only identified at the phylum level, and despite the importance of this phylum for aquatic environments, current knowledge about their specific ecological role is still very limited.

The co-occurrence network exhibited a moderate to low level of modularity, but within these modules, the nodes were highly connected. Additionally, we observed nodes that were unique to specific areas and others that were shared (Figure 6). When we observed the OTUs commonly found across all areas, they were mainly composed of fungi from the phyla Ascomycota and Basidiomycota (Table S2), with the most abundant and prevalent genus being versatile immigrant fungi. These two groups represent the main saprophytic soil fungal decomposers traditionally identified in different soil niches [65]. Nevertheless, from a functional perspective, the degradation of complex and recalcitrant organic matter, such as lignocellulose, is generally considered to be limited to Basidiomycota [66,67]. In addition to their decomposer role, Ascomycota also encompasses both plant and animal pathogens, as well as species that offer notable benefits such as sources of medicine or hold significant economic importance, including yeasts. Both Ascomycota and Basidiomycota can colonize different substrates, resulting in a cosmopolitan distribution that extends to include aquatic environments. This broad distribution may help explain the co-occurrence of these fungi in all locations within the lagoon. The residential area and estuarine mouth exhibited the highest number of unique OTUs. When we observed these unique OTUs, although they comprised fungi from the phyla Ascomycota and Basidiomycota, they also represented a substantial proportion of unidentified fungi and fungi from the phylum Chytridiomycota (Table S3). The residential area and estuarine mouth were in locations that formed ecotones, which were characterized by the coexistence and interaction of multiple communities [21]. Consequently, a diverse range of environmental conditions emerges, capable of supporting species absent in neighboring habitats. Nonetheless, although the marine and anthropogenic inputs in these lacustrine environments can lead to the introduction of numerous species, many of these species may lack the necessary adaptations to survive in such an environment. Consequently, while they may contribute to the local species diversity when analyzed using DNA techniques, it is crucial to acknowledge that a significant number of them will not possess the adaptations required for their survival. Thus, it is worth noting that metabarcoding analyses cannot differentiate between living and deceased organisms, which is an important limitation that needs to be considered when employing environmental DNA techniques.

Our study was conducted during a low tide period, when the Tramandaí Lagoon was experiencing the ebbing tide. The lagoon is a highly dynamic environment that can be influenced by tidal fluctuations, which result in the influx of seawater through the estuarine channel [25], variations in river water levels, and the quantity of waste discharged into the lagoon. Although this can influence water circulation patterns and, consequently, the fungal community of the lagoon, we believe that the low tide conditions provide the opportunity to create distinct environments that are more affected by the local surroundings and nearby effluents. This can lead to the formation of suitable habitats for specific organisms. This seemed to be corroborated by the fact that we observed some unique OTUs in only certain locations. Thus, during low tide periods, water circulation within the lagoon may not exert a significant influence on the community structure and composition. On the other hand, during high tide, it is possible that increased water circulation leads to greater homogenization of these distinct environments. This phenomenon arises from the amplified water mixing and increased fungal dispersal between environments. Consequently, fungi across different locations may exhibit greater similarity, significantly impacting the overall structure and composition of the fungal community. In addition, with the increased influx of seawater into the lagoon, there is a rise in local salinity. This can also lead to species homogenization, as not all organisms are capable of thriving in environments with elevated salinity levels. However, it is important to highlight that the predominant liquid flow in the lagoon is towards the sea, but, obviously, it is also possible to observe opposite flows [68]. Generally, the coastal area is characterized by small tidal effects [68]. In the future, it would be interesting to test this hypothesis related to the tide level to provide insight into how water circulation in a lagoon under various influences can affect the structure and composition of the fungal community.

All of the environmental variables (pH, conductivity, dissolved oxygen, biological oxygen demand), with the exception of water temperature, were associated with the sampling units of the estuary, middle of the lagoon, and crop field area. Moreover, the middle of the lagoon and crop field area were much more homogeneous regarding both mycobiome diversity and physicochemical characteristics than the estuary, which, in turn, exhibited a high diversity of unique taxa. Conversely, the residential area was associated with water temperature and was highly divergent from the other areas, considering both fungal diversity and physicochemical features. This difference may occur because this area receives sewage from a residential region, which generates eutrophication that can lead to an unbridled increase in fungal biomass, especially of species that feed on decomposing organic matter. For instance, the decomposition of leaves was on average 2.3–2.7× faster in eutrophicated streams than in non-eutrophicated streams due to the stimulation of fungal activity by dissolved nutrients [9]. Furthermore, in this area, the water temperature was sampled in a marginal zone, which was slightly shallower. Hence, this area was subjected to higher radiation incidence compared to the other areas, which may have been influenced by the higher temperature at this point. Nonetheless, the magnitude of the difference between the points was very small, and it is unlikely that 1 or 2 degrees in temperature caused divergence in terms of physicochemical characteristics (Table S4). Therefore, we believe that this difference is due to the contribution from the margin combined with specific local conditions (effluents from residential areas jointly with effluents from the Tramandaí River). In addition, we observed that this point also displayed a lower BOD. BOD is a parameter widely used to assess organic pollution in water systems and is inversely related to fungal diversity [51,69]. It is important to highlight that the estuary area receives sewage from the residential area, leading to the availability of large amounts of organic matter that can favor the abundance of certain groups of fungi while inhibiting the presence of others. Nevertheless, this area also benefits from the Tramandaí River, which seems to promote the diversity of fungi in those locations. Thus, freshwater input from the Tramandaí River into the lagoon likely influences the BOD and other physicochemical parameters of the water, ultimately contributing to the higher fungal diversity observed at this specific point in the lagoon.

## 5. Conclusions

To sum up, our study revealed that in the ecotonal areas in the Tramandaí Lagoon, represented by estuary (lagoon/ocean) and residential (lagoon/river) zones, the fungal communities were significantly more diverse than in the two non-ecotonal areas (middle of the lagoon and crop field area) and both shared and unique fungal taxa that exhibited a high proportion of eDNA from immigrant terrestrial fungi. However, the effects of river inflow on the lagoon appeared to be greater than those of sewage. Further studies are needed to understand the effects of these anthropogenic impacts on the fungal community. In addition to Ascomycota and Basidiomycota, Chytridiomycota (*s.l.*) were dominant, and most of the chytridiomycotan genera were largely unknown since they were mainly identified only at higher level taxonomic categories. This scenario also extended to ascomycotan and basidiomycotan fungal taxa. Therefore, our findings point out that, even using a metabarcoding approach, a considerable proportion of completely unknown fungal taxa still exists. Additionally, further research is necessary to fully comprehend the role of unidentified or partially identified fungi and their response to both environmental and anthropogenic impacts.

## Figures and Tables

**Table 1:** Fungal taxa and their corresponding main mode of nutrition, ecological guild, and body morphology (Rhizomycelial: fungi whose body structure is a rhizomycelium) identified at the genus level in the superficial water of Tramandaí Lagoon. Information obtained from FUNGuild [37]. ‘na’ represents information not found.

Genus	Trophic Mode	Ecological Guild	Body Morphology
ASCOMYCOTA			
*Alternaria*	Pathotroph-Saprotroph-Symbiotroph	Animal Pathogen-Endophyte-Plant Pathogen-Wood Saprotroph	Mycelial
*Apiospora*	Pathotroph-Saprotroph	Plant Pathogen	Mycelial
*Aureobasidium*	Pathotroph-Saprotroph-Symbiotroph	Animal Pathogen-Endophyte-Epiphyte-Plant Pathogen-Undefined Saprotroph	Yeast
*Cercospora*	Pathotroph	Plant Pathogen	Mycelial
*Clonostachys*	Pathotroph	Plant Pathogen	Mycelial
*Colletotrichum*	Pathotroph-Symbiotroph	Endophyte-Plant Pathogen	Mycelial
*Coniella*	Pathotroph	Plant Pathogen	Mycelial
*Curvularia*	Pathotroph	Plant Pathogen	Mycelial
*Diaporthe*	Pathotroph-Symbiotroph	Endophyte-Plant Pathogen	Mycelial
*Erysiphe*	Pathotroph	Plant Pathogen	Mycelial
*Exserohilum*	Pathotroph	Plant Pathogen	Mycelial
*Fusarium*	Pathotroph-Saprotroph-Symbiotroph	Animal Pathogen-Endophyte-Lichen Parasite-Plant Pathogen-Soil Saprotroph-Wood Saprotroph	Mycelial
*Gliomastix*	Saprotroph	Undefined Saprotroph	Mycelial
*Lophiostoma*	Saprotroph	Animal Pathogen	Mycelial
*Metarhizium*	Pathotroph	na	Mycelial
*Meyerozyma*	na	na	Yeast
*Microdochium*	na	na	Mycelial
*Nannizzia*	na	na	Mycelial
*Neodevriesia*	na	na	Mycelial
*Neopyrenochaeta*	na	Undefined Saprotroph	Mycelial
*Neurospora*	Saprotroph	Undefined Saprotroph	Mycelial
*Niesslia*	Saprotroph	Animal Pathogen	Mycelial
*Nigrograna*	Pathotroph	Undefined Saprotroph	Mycelial
*Nigrospora*	Saprotroph	na	Mycelial
*Omnidemptus*	na	na	Mycelial
*Paracylindrocarpon*	na	Dung Saprotroph-Undefined Saprotroph-Wood Saprotroph	Mycelial
*Penicillium*	Saprotroph	Endophyte-Plant Pathogen-Wood Saprotroph	Mycelial
*Periconia*	Pathotroph-Saprotroph-Symbiotroph	Plant Pathogen	Mycelial
*Phaeoacremonium*	Pathotroph	Endophyte-Plant Pathogen	Mycelial
*Plectosphaerella*	Pathotroph-Symbiotroph	Plant Pathogen	Mycelial
*Pseudocercospora*	Pathotroph	na	Mycelial
*Pseudopyricularia*	na	Endophyte-Lichen Parasite-Undefined Saprotroph	Mycelial
*Pyrenochaetopsis*	Pathotroph-Saprotroph-Symbiotroph	na	Mycelial
*Ramichloridium*	na	Undefined Saprotroph	Mycelial
*Saccharomyces*	Saprotroph	na	Yeast
*Striaticonidium*	na	Undefined Saprotroph	Mycelial
*Talaromyces*	Saprotroph	Undefined Saprotroph	Mycelial
*Teichospora*	Saprotroph	Endophyte-Plant Pathogen	Mycelial
*Toxicocladosporium*	Pathotroph-Symbiotroph	Animal Pathogen-Endophyte-Epiphyte-Fungal Parasite-Plant Pathogen-Wood Saprotroph	Mycelial
*Trichoderma*	Pathotroph-Saprotroph-Symbiotroph	Endophyte	Mycelial
*Trichomerium*	Symbiotroph	Undefined Saprotroph	Mycelial
*Uwebraunia*	Saprotroph	na	Mycelial
*Zymoseptoria*	na		Mycelial
BASIDIOMYCOTA			
*Agaricus*	na	na	Mycelial
*Amanita*	Saprotroph-Symbiotroph	Ectomycorrhizal-Undefined Saprotroph	Mycelial
*Atractidochium*	na	na	Mycelial
*Chaetospermum*	na	na	Mycelial
*Cintractia*	Pathotroph	Plant Parasite-Plant Pathogen	Mycelial
*Coprinellus*	Saprotroph	Undefined Saprotroph	Mycelial
*Crustoderma*	Saprotroph	Wood Saprotroph	Mycelial
*Curvibasidium*	na	na	Yeast
*Erythrobasidium*	na	na	Yeast
*Farysia*	Pathotroph	Plant Pathogen	Yeast
*Fellomyces*	na	na	Yeast
*Hannaella*	na	na	Yeast
*Kockovaella*	Symbiotroph	Epiphyte	Yeast
*Limonomyces*	Pathotroph	Plant Pathogen	Mycelial
*Lyomyces*	na	na	Mycelial
*Malassezia*	Pathotroph-Saprotroph	Animal Pathogen-Undefined Saprotroph	Yeast
*Marchandiomyces*	Pathotroph	Lichen Parasite	Mycelial
*Meira*	na	na	Yeast
*Moesziomyces*	Pathotroph	Plant Pathogen	Yeast
*Naganishia*	na	na	Yeast
*Papiliotrema*	na	na	Yeast
*Peniophora*	Pathotroph-Saprotroph	Plant Pathogen-Wood Saprotroph	Mycelial
*Peniophorella*	Saprotroph	Undefined Saprotroph	Mycelial
*Phellinus*	Pathotroph-Saprotroph	Plant Pathogen-Wood Saprotroph	Mycelial
*Phlebiopsis*	Saprotroph	Undefined Saprotroph	Mycelial
*Pseudohyphozyma*	na	na	Yeast
*Pseudomicrostroma*	na	na	Yeast
*Resinicium*	Saprotroph	Undefined Saprotroph	Mycelial
*Rhizopogon*	Symbiotroph	Ectomycorrhizal	Mycelial
*Rhodotorula*	Pathotroph-Saprotroph	Animal Endosymbiont-Animal Pathogen-Endophyte-Plant Pathogen-Undefined Saprotroph	Yeast
*Saitozyma*	na	na	Yeast
*Schizophyllum*	Saprotroph	Wood Saprotroph	Mycelial
*Sterigmatomyces*	na	na	Yeast
*Suillus*	Symbiotroph	Ectomycorrhizal	Mycelial
*Trechispora*	Saprotroph	Wood Saprotroph	Mycelial
*Tritirachium*	Saprotroph	Undefined Saprotroph	Mycelial
*Vishniacozyma*	na	na	Yeast
*Wallemia*	Saprotroph	Undefined Saprotroph	Yeast
*Xylodon*	Saprotroph	Undefined Saprotroph	Mycelial
CHYTRIDIOMYCOTA			
*Entoplyctis*	na	na	Rhizomycelial
*Rhizoplyctis*	na	na	Rhizomycelial

## Data Availability

The data presented in this study are available in Tables S1, S3 and S4.

## Supplementary Materials

The following supporting information can be downloaded at: https://www.mdpi.com/article/10.3390/jof9090890/s1, Table S1: Metadata of 1930 putative species (OTUs) and their abundances at each of the 4 points registered in the Tramandaí Lagoon, Brazil. Figure S1: Maximum likelihood phylogenetic tree using the 1930 OTUs identified in all sampling points, generated with 1000 bootstrap replicates. Figure S2: Co-occurrence network of putative species (OTUs) in the sampling areas (estuary, middle of the lagoon, residential area, and crop field area). Figure S3: Canonical correspondence analysis demonstrating that the physicochemical variables influence species composition. Table S2: Metadata of co-occurrence of putative species (OTUs) in the Tramandaí Lagoon, Brazil. Table S3: Metadata of co-occurrence of the 10% most dominant OTUs. Table S4: Physicochemical features of distinct sampling areas in Tramandaí Lagoon, Brazil.
