# Supplementary material for: Unveiling Fungal Community Structure along Different Levels of Anthropic Disturbance in a South American Subtropical Lagoon
Source: J Fungi (Basel). 2023 Aug 31;9(9):890. doi: 10.3390/jof9090890 (PMC10532596; doi:10.3390/jof9090890)
Supplement: Supplementary file 1 [file jof-09-00890-s001.zip › TableS4_Physicochemical_variables.pdf]

Table S4: Physicochemical features of distinct sampling areas in Tramandaí Lagoon (Brazil).

Sampling Points/ Features	Estuary (P1)	Middle Lagoon (P2)	Cops fields (P3)	Residential (P4)
BOD (mg/L)	1.34	1.92	1.73	0.96
OD (mg/L)	7.49	7.87	7.10	6.53
pH	7.82	7.77	7.68	7.53
Chlorides (mg/L)	6626.70	4597.83	4487.04	754.77
Salinity (‰)	11.97	8.31	8.11	1.36
Conductivity (µs/cm)	1874.00	13000.00	12730.00	1690.00
Water Temperature (°C)	24	23	23	25
