# Supplementary figures and images for: Unveiling Fungal Community Structure along Different Levels of Anthropic Disturbance in a South American Subtropical Lagoon
Source: J Fungi (Basel). 2023 Aug 31;9(9):890. doi: 10.3390/jof9090890 (PMC10532596; doi:10.3390/jof9090890)

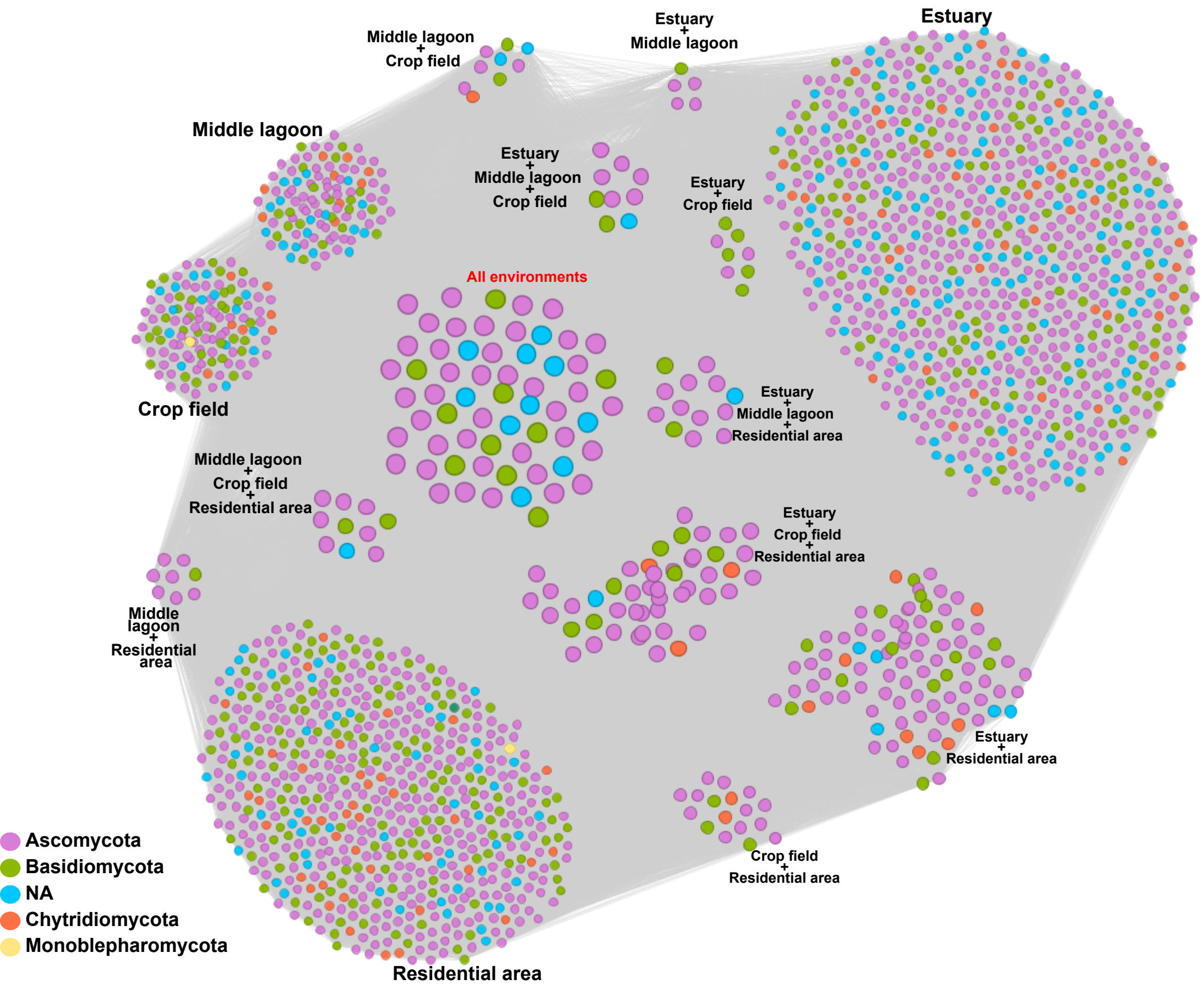

Supplement: Supplementary file 1 [file jof-09-00890-s001.zip › FigureS2_network.pdf]

Axis 2

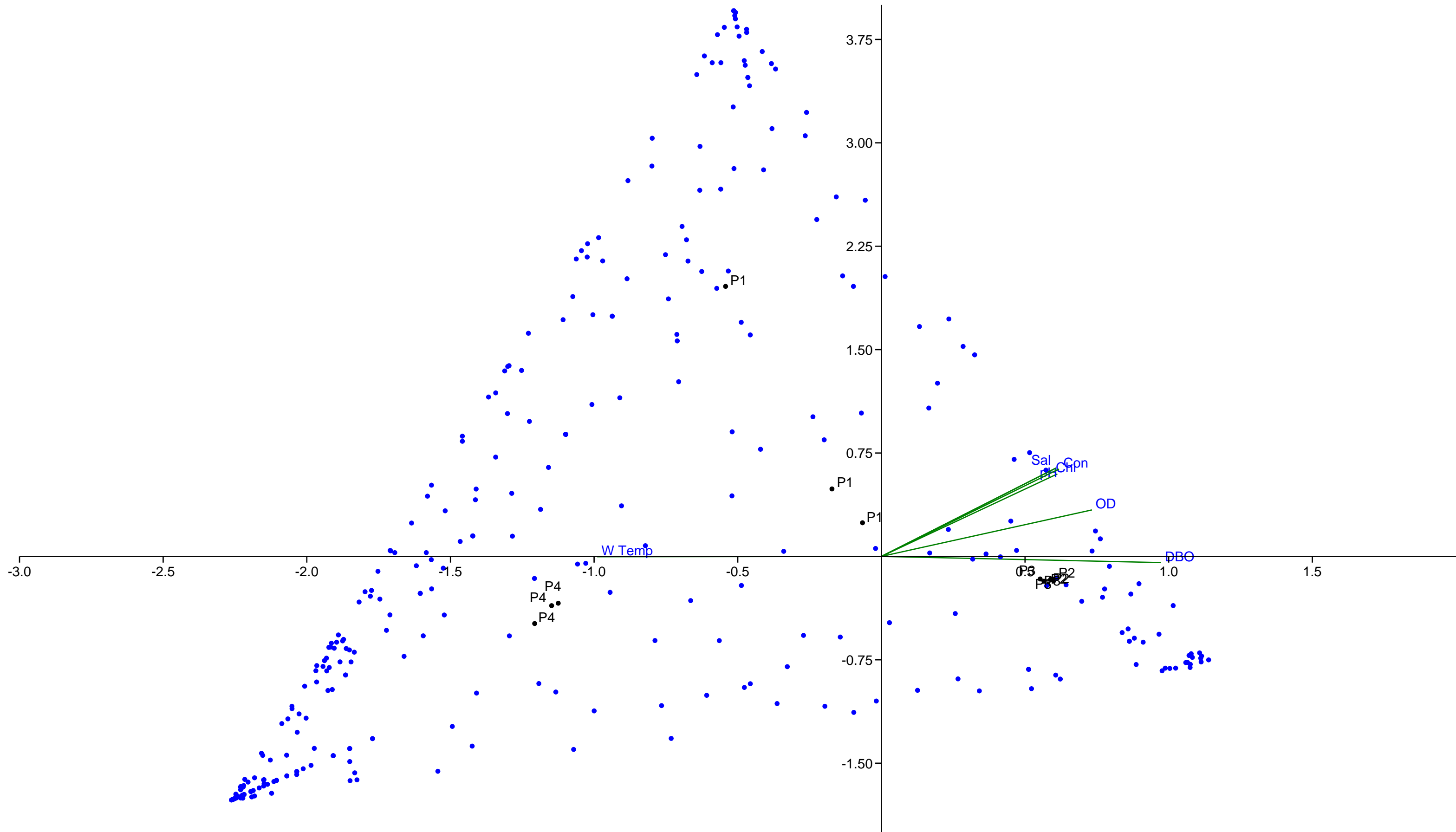

Axis 1

Supplement: Supplementary file 1 [file jof-09-00890-s001.zip › FigureS3_CCA.pdf]
