# Supplementary material for: Unveiling Fungal Community Structure along Different Levels of Anthropic Disturbance in a South American Subtropical Lagoon
Source: J Fungi (Basel). 2023 Aug 31;9(9):890. doi: 10.3390/jof9090890 (PMC10532596; doi:10.3390/jof9090890)
Supplement: Supplementary file 1 [file FigureS1_tree_allOTU_its.pdf › FigureS1_tree_allOTU_its.pdf]

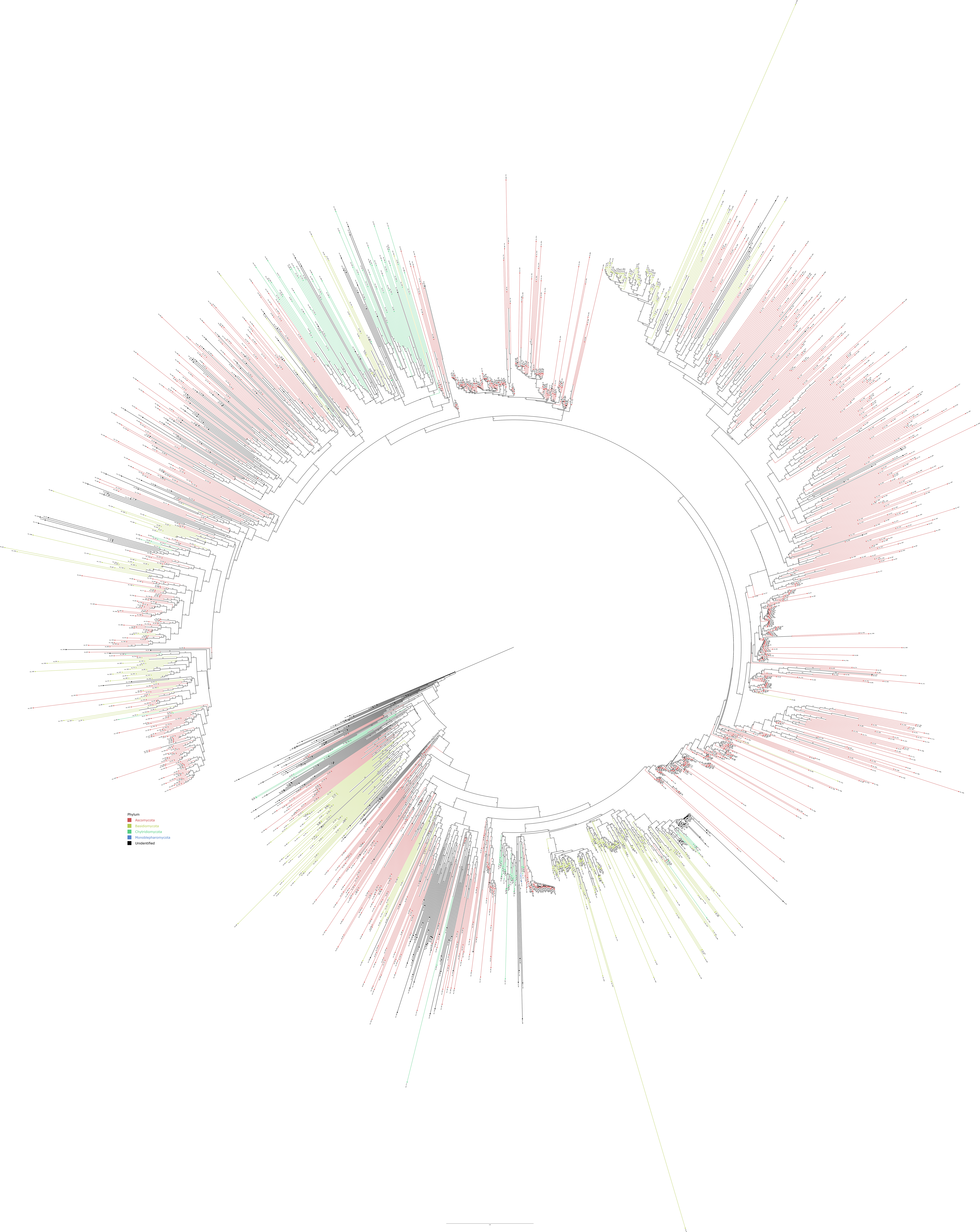
Phylum
Ascomycota
Basidiomycota
Chytridiomycota
Monoblepharomycota
Unidentified
